# Association of Obesity Indicators with Gestational Diabetes: A Comparative Study from NHANES

**DOI:** 10.2174/0118715303466539260525105816

**Published:** 2026-06-10

**Authors:** Jing Tang

**Affiliations:** 1 Shanghai Key Lab of Female Reproductive Endocrine Related Diseases, Obstetrics & Gynecology Hospital of Fudan University, Shanghai Key Lab of Reproduction and Development, Shanghai, China

**Keywords:** Gestational diabetes mellitus, obesity indicators, relative fat mass, risk screening, National Health and Nutrition Examination Survey (NHANES)

## Abstract

**Introduction:**

This study aims to explore the relationships between various obesity-related indicators and gestational diabetes mellitus (GDM), as well as assess their ability to predict GDM development.

**Materials and Methods:**

Data from the 2007–2020 National Health and Nutrition Examination Survey included 4,000 women with either a history of live birth or a gestational age of at least 28 weeks. We used weighted multivariable logistic regression to examine the links between nine obesity indicators—such as body roundness index (BRI), relative fat mass (RFM), lipid accumulation product (LAP), visceral adiposity index (VAI), waist-to-height ratio (WHtR), weight-adjusted waist index (WWI), Conicity Index, body shape index (ABSI), and body mass index (BMI)—and GDM. To explore potential nonlinear relationships, restricted cubic splines were employed, and receiver operating characteristic (ROC) curves were used to evaluate and compare the predictive accuracy of each measure.

**Results:**

Among participants, 352 individuals (8.8%) were diagnosed with GDM. There were positive correlations between GDM risk and RFM, BRI, VAI, LAP, WHtR, WWI, Conicity Index, and BMI, while ABSI showed no significant correlation. Nonlinear analysis indicated threshold effects for VAI, LAP, and BMI concerning GDM.

**Discussion:**

RFM demonstrated the strongest predictive ability with the highest area under the curve (0.690; 95% CI: 0.662–0.717). Subgroup analysis revealed an interaction between alcohol consumption and the relationship between RFM and GDM (*P* = 0.039).

**Conclusion:**

RFM showed the most consistent association with GDM and achieved the best predictive performance, making it a promising candidate for clinical screening of women at high risk for GDM.

## 1 INTRODUCTION

Gestational diabetes mellitus (GDM) is marked by impaired glucose tolerance that appears or is diagnosed during pregnancy, making it one of the most common pregnancy complications [1, 2]. Globally, the prevalence of GDM has been increasing annually, significantly threatening maternal and fetal health. It is linked to negative pregnancy outcomes such as gestational hypertension, cesarean section, and preterm birth [3]. Furthermore, GDM has been proven to markedly elevate the long-term risk of type 2 diabetes, cardiovascular disease, and metabolic syndrome in both mothers and their children [1, 2, 4]. Therefore, it is crucial for public health to identify high-risk groups for GDM and to explore its modifiable risk factors. These efforts can support the development of early prevention and intervention strategies aimed at enhancing long-term health outcomes for the population.

Obesity is a recognized risk factor for GDM [1, 5, 6]. Conventional metrics like body mass index (BMI) are commonly used in clinical and public health contexts because they are simple and easy to apply. However, BMI has shortcomings in distinguishing fat from muscle and cannot precisely indicate body fat distribution, particularly visceral fat, which is strongly associated with metabolic disease risk. To better assess obesity and related health risks, new indicators have been developed. These combine factors such as waist circumference, height, weight, and blood lipids to enhance the measurement of fat distribution and visceral fat buildup. Examples include the lipid accumulation product (LAP) and the visceral adiposity index (VAI), which incorporate waist circumference and blood lipid levels, and are effective in reflecting visceral obesity and insulin resistance [7, 8]. The body roundness index (BRI) and the relative fat mass (RFM) employ geometric models or simplified formulas to more accurately estimate body fat percentage [9, 10]. Additional metrics have been created to evaluate body shape, with the weight-adjusted waist index (WWI) standing out as a key example. By accounting for body weight, the WWI offers a more consistent measure of central obesity [11, 12]. The waist-to-height ratio (WHtR) is a simple yet effective screening tool for central obesity [13]. The Conicity Index evaluates the degree of deviation from a cylindrical body shape [14]. The body shape index (ABSI) provides a BMI-independent predictor of mortality by incorporating height, weight, and waist circumference [15]. While these novel metrics show superior predictive value in chronic diseases such as cardiovascular disease and type 2 diabetes [16-22], systematic comparative studies evaluating them in the context of GDM are scarce.

In recent years, an increasing number of studies have focused on the role of novel obesity indicators in assessing women's metabolic health and the risk of pregnancy complications. In a Brazilian study, Rangel *et al*. compared the predictive abilities of BRI, RFM, and traditional anthropometric measures (BMI, waist circumference) for body fat, insulin resistance, and metabolic syndrome. The findings indicated that while conventional metrics demonstrated overall effectiveness, BRI outperformed BMI and waist-to-hip ratio in predicting metabolic syndrome, underscoring its potential as a clinical adjunct tool [23]. Another study confirmed that RFM outperformed BMI in identifying dyslipidemia and metabolic syndrome, suggesting that it better reflects the fat burden associated with metabolic abnormalities [24]. Furthermore, an association between dietary inflammation indices and type 2 diabetes risk was found in women with a history of GDM, RFM, and BMI, indicating that obesity measures also play a crucial role in long-term metabolic management post-GDM [25]. Preliminary research indicates that elevated LAP levels in the early stages of pregnancy are strongly associated with insulin resistance and impaired glucose regulation in mid-pregnancy. This suggests that LAP may serve as an early screening marker for gestational glucose metabolism disorders [26]. In women diagnosed with polycystic ovary syndrome, the severity of VAI and LAP has been demonstrated to be closely associated with the presence of insulin resistance and metabolic syndrome, thereby exhibiting satisfactory predictive efficacy [27]. Recent research suggests that novel obesity indicators, such as BRI, RFM, VAI, and LAP, may offer advantages for evaluating insulin resistance, metabolic syndrome, and GDM risk. However, systematic comparative studies evaluating the predictive capacity of these novel indicators *versus* traditional BMI in large, representative populations remain scarce. The National Health and Nutrition Examination Survey (NHANES), a nationally representative database with extensive anthropometric, laboratory, and reproductive health data, provides an ideal platform that systematically addresses this issue. Therefore, the objective of this study is to utilize NHANES data from 20072020 cycles to investigate associations between nine obesity-related indicators (LAP, VAI, BRI, RFM, WWI, WHtR, Conicity Index, ABSI, and BMI) and GDM history in a nationally representative sample of U.S. adult women. The significance of these indicators in assessing GDM risk will be examined using various statistical techniques, such as multivariate adjustment models, exploring nonlinear relationships, comparing predictive performances, and conducting subgroup analyses. This approach will offer stronger evidence-based support for early GDM detection and risk stratification.

## 2 MATERIALS AND METHODS

### 2.1 Study Population

Data were collected from NHANES, a nationally representative public database managed by the Centers for Disease Control and Prevention. NHANES uses a complex multistage probability sampling design and releases data every two years. This study integrated data from seven consecutive cycles spanning from 2007 to 2020 (2007–2008, 2009–2010, 2011–2012, 2013–2014, 2015–2016, 2017–2020). The research protocol for NHANES received approval from the National Center for Health Statistics Ethics Review Board, and all participants gave written informed consent.

The initial study population consisted of women who had either at least one previous live birth or were currently pregnant at 28 weeks or more. Exclusions applied to individuals with missing data on critical obesity indicators, incomplete GDM questionnaires, or missing information on key covariates such as age at interview, race, education level, poverty income ratio (PIR), marital status, smoking, alcohol intake, hypertension, depression, physical activity, having an infant with a birth weight of 9 pounds or more, or age at first live birth. After exclusions, 4,000 participants were included in the analysis. The participant selection process is delineated in Fig. (**1**).

### 2.2 Definition of GDM

GDM diagnosis was determined based on participants' self-reported responses to the reproductive health questionnaire: “During pregnancy, were you ever told by a doctor or other health professional that you had diabetes, sugar diabetes, or gestational diabetes? Please do not include diabetes that you may have known about before the pregnancy.” Affirmative responses indicated a history of GDM [28].

### 2.3 Definition of Obesity-Related Indicators

Nine obesity indicators were used as exposure variables: LAP, BRI, VAI, RFM, WWI, WHtR, Conicity Index, ABSI, and BMI. The calculation formulas for each indicator are as follows:

1. LAP = (WC(cm) - 65) × TG (mmol/L) in males and LAP = (WC(cm) - 58) × TG (mmol/L) in females [7]
2. BRI = 364.2 - 365.5 x 
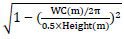
 [9]
3. VAI = 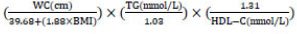 in males and VAI = 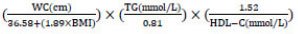 in females [8]
4. RFM=64-(20 × (height(m)⁄WC(m))) + (12 × sex), sex = 0 for male and 1 for female [10]
5. WWI = 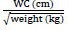 [11]
6. WHtR = WC (cm)⁄Height (cm) [13]
7. Conicity index = 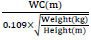 [14]
8. ABSI = 
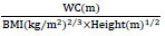
 [15]
9. BMI = Weight (Kg)⁄(Height^2^ (m^2^))

To optimize the analysis and achieve a more precise evaluation of the association between ABSI and GDM, a standardization process was implemented on ABSI, resulting in the generation of its Z-score, which is herein designated as ABSI^a^. Continuous obesity indicators were categorized into tertiles (T1, T2, T3), with T1 serving as the reference group.

### 2.4 Assessment of Covariates

The following covariates were considered in this study: age at interview, age at first live birth, race, PIR, education level, marital status, smoking status, alcohol consumption status, hypertension, depression, physical activity, and whether the infant's birth weight was ≥9 pounds. Participants were categorized into five racial groups: Mexican American, non-Hispanic White, non-Hispanic Black, other Hispanic, and other races. Educational attainment was grouped into three categories: less than high school, high school or equivalent, and beyond high school. Marital status of the participants was classified into three categories: married/cohabiting, never married, and widowed/divorced/separated. Smoking status was determined using standard questionnaires, with participants categorized as never smokers (lifetime smoking <100 cigarettes), former smokers (≥100 cigarettes smoked but currently abstinent), or current smokers. Alcohol consumption was classified according to participant reports of “at least 12 alcoholic drinks per year,” with any missing data recorded as “unknown.” Hypertension was defined as a systolic blood pressure of ≥130 mmHg, a diastolic blood pressure of ≥85 mmHg, or current use of antihypertensive medication. Depression severity was assessed using the Patient Health Questionnaire-9 (PHQ-9), where a total score of ≥10 indicated depressive symptoms [29]. Physical activity was divided into two groups: “meeting guidelines” and “not meeting guidelines.” These groups were defined by whether the individual achieved at least 600 MET-minutes per week, which is the recommended level.

### 2.5 Statistical Analysis

To account for the complex sampling design of NHANES, all analyses included sample weights, clustering, and stratification variables, ensuring the results are statistically valid and representative of the U.S. population. Baseline characteristics were thoroughly described, clearly distinguishing between participants with and without GDM. Continuous variables that met the normality assumption were reported as mean ± standard deviation, with comparisons between groups performed using weighted t-tests. Non-normally distributed continuous variables were presented as median (interquartile range (IQR)), with group comparisons conducted *via* the Kruskal-Wallis H test. Categorical variables were expressed as weighted frequencies (percentages), and intergroup differences were tested using weighted chi-square tests or Fisher's exact tests.

Multicollinearity was evaluated with the variance inflation factor (VIF), and all VIF values for the included covariates were below 5, suggesting no significant collinearity issues. Weighted multivariable logistic regression models, implemented with the R `survey` package, examined the associations between obesity measures and GDM. Three models were created: Model 1 (unadjusted, no covariates), Model 2 (adjusted for demographic variables such as age at interview, ethnicity, education level, PIR, and marital status), and Model 3 (further adjusted for lifestyle and health factors including smoking, alcohol use, hypertension, depression, physical activity, birth weight ≥9 lbs [macrosomic birth], and age at first live birth). Nonlinear relationships between obesity indicators and GDM were explored using restricted cubic splines (RCS). Specifically, we employed three knots positioned at the 10^th^, 50^th^, and 90^th^ percentiles. This approach ensured curve flexibility while preventing overfitting, contributing to the model's overall effectiveness. The model’s predictive performance was evaluated by plotting ROC curves and calculating the AUC. Subgroup analyses and interaction tests assessed heterogeneity across different groups.

To ensure robustness, a series of sensitivity analyses was carried out. The primary analyses were rerun with unweighted data. Furthermore, Model 3 was progressively adjusted for factors such as vaginal delivery frequency, contraceptive use, female hormone use, and the number of live births. Lastly, we applied multiple imputation techniques to address missing covariates and performed association analyses on the imputed datasets. All analyses were conducted using R software (version 4.4.1), with a P value of less than 0.05 indicating statistical significance.

## 3 RESULTS

### 3.1 Baseline Characteristics of the Study Population

The final sample included 4,000 participants, with 352 (8.8%) reporting a history of GDM. Baseline characteristics are outlined in Table **1**. The median age at first live birth was 22 years (IQR: 19–26 years). Depression prevalence was 10.56%, and hypertension prevalence of hypertension was 44.97%, with 19.13% of participants reporting a history of macrosomic birth (≥9 lbs). Compared to the non-GDM group, GDM participants had significantly higher prevalence of depression, macrosomic birth history, and elevated levels of RFM, BRI, LAP, VAI, WHtR, waist circumference, weight, and BMI (all *p* < 0.05). They also had lower interview age and HDL-cholesterol levels (all *p* < 0.05).

### 3.2 Association Analysis of Obesity Indicators with GDM

Weighted multivariable logistic regression was employed to evaluate the associations between nine obesity-related indicators and the risk of GDM across three sequential models. In the unadjusted analysis (Model 1), continuous measures of RFM, BRI, VAI, LAP, WHtR, WWI, and BMI all showed significant positive associations with GDM risk. These associations persisted and were generally strengthened after incremental adjustment for demographic covariates (Model 2) and further for lifestyle and clinical factors (Model 3). In the fully adjusted model (Model 3), most obesity indicators remained independently and significantly associated with increased GDM odds. The per-unit increase in continuous RFM was associated with a 5% higher risk of GDM (OR = 1.05, 95% CI: 1.02–1.09, *p* = 0.002). Similarly, significant positive associations were observed for BRI (OR = 1.10, 95% CI: 1.04–1.16, *p* < 0.001), VAI (OR = 1.07, 95% CI: 1.02–1.13, *p* = 0.009), LAP (OR = 1.42, 95% CI: 1.14–1.76, *p* = 0.002), WHtR (OR = 11.7, 95% CI: 2.76–49.8, *p* = 0.001), WWI (OR = 1.42, 95% CI: 1.20–1.69, *p* < 0.001), Conicity Index (OR = 1.25, 95% CI: 1.09–1.44, *p* = 0.002), and BMI (OR = 1.02, 95% CI: 1.00–1.05, *p* = 0.024). Categorical analysis revealed significant associations between the third tertile (T3) of most indicators and the risk of GDM. (*e.g.*, RFM T3 OR = 1.71, 95% CI: 1.14–2.58, *p* = 0.010). Conversely, ABSI^a^ showed no significant association in any model (all *p* > 0.05) (Table **2**).

### 3.3 RCS Analysis of the Relationship Between Obesity-related Indicators and GDM

The RCS model was used to examine the dose-response relationship between obesity indicators and GDM. The results (Fig. **2**) indicated that, after controlling for demographic, lifestyle, and clinical factors, several obesity-related markers exhibited complex dose-response relationships with GDM risk. VAI, LAP, and BMI showed nonlinear associations (nonlinear *p*-values < 0.05), with curves featuring notable inflection points. This implies a nonlinear link between GDM risk and these indicators, with a more rapid increase within certain ranges. In contrast, BRI, RFM, WWI, WHtR, and the Conicity Index displayed a straightforward linear positive relationship with GDM (nonlinear *p*-values > 0.05). These findings highlight the need to account for potential nonlinear effects when assessing how obesity indicators influence GDM risk.

### 3.4 Predictive Performance Evaluation of Obesity-related Indicators

The predictive accuracy of various obesity-related indicators for GDM was assessed with ROC curves and AUC metrics (Fig. **3** and Table **3**). The AUC for BMI was 0.681 (95% CI: 0.654–0.708). For RFM, the AUC was 0.690 (95% CI: 0.662–0.717), showing a significant difference from BMI (*p* < 0.001). Among the indicators, BRI (AUC = 0.685, 95% CI: 0.658–0.712, *p* = 0.036) and WHtR (AUC = 0.687, 95% CI: 0.659–0.714, *p* = 0.006) had notably higher AUC values than BMI. The AUCs for other models were: WWI (AUC = 0.689, p = 0.082), Conicity Index (AUC = 0.685, *p* = 0.289), LAP (AUC = 0.687, *p* = 0.114), and VAI (AUC = 0.687, *p* = 0.202). Although these values are slightly higher, they did not reach statistical significance. ABSI^a^ showed an AUC of 0.680, with no statistically significant difference compared to BMI (*p* = 0.798). Overall, RFM, BRI, and WHtR demonstrated better discriminatory ability for GDM prediction.

### 3.5 Subgroup Analysis of the Relationship Between RFM and GDM

Stratified analyses examined RFM and GDM associations across subgroups (Table **4**). In the racial subgroup, both non-Hispanic Black (OR = 1.06, 95% CI: 1.00–1.13, *p* = 0.034) and non-Hispanic White (OR = 1.06, 95% CI: 1.01–1.11, *p* = 0.021) showed significant links between RFM and GDM risk. Nonetheless, no significant interaction effect across racial groups was found (*p* for interaction = 0.649). In the education subgroup, RFM had the strongest association with GDM among individuals with more than a high school education (OR = 1.07, 95% CI: 1.03–1.11, *p* < 0.001). However, the education interaction was not statistically significant (*p* = 0.301). Married/cohabiting participants showed notable associations (OR = 1.07, 95% CI: 1.02–1.11, *p* = 0.002). Former smokers (OR = 1.09, 95% CI: 1.01–1.17, *p* = 0.027) and never smokers (OR = 1.07, 95% CI: 1.04–1.11, *p* < 0.001) showed significant correlations, with no significant interaction for smoking status (*p* = 0.098). Alcohol consumers exhibited a significant association (OR = 1.09, 95% CI: 1.04–1.14, *p* < 0.001) and interaction (*p* = 0.039). Associations between RFM and GDM with the other subgroups (physical activity, depression, history of macrosomia, and hypertension) were significant to varying degrees, but the interactions were not significant (*p* > 0.05).

### 3.6 Sensitivity Analysis

Sensitivity analyses confirmed robustness. Unweighted data were concordant with the weighted analysis results, with unchanged direction and significance for significant associations (Supplementary Material, Table **S1**). After comprehensive adjustments of Model 3 with subsequent control for potential confounders, including the number of vaginal deliveries, contraceptive use, female hormone use, and number of live births, associations between all obesity indicators and GDM remained significant, and the core conclusions were consistent (Supplementary Material, Table **S2**). Analysis after multiple imputation indicated that the main findings remained largely consistent with the results from the unimputed data analysis, with obesity-related indicators continuing to show a significant positive association with GDM (Supplementary Material, Table **S3**).

## 4 DISCUSSION

This cross-sectional study, which used a nationally representative NHANES sample, systematically evaluated and compared the relationships between nine different obesity-related indicators and a history of GDM. It’s important to highlight that these findings show associations, not causation, which is a key limitation due to the study's design. The main contribution is its comprehensive initial comparison of both new and traditional adiposity measures with GDM risk in a large, population-based U.S. cohort. After adjusting for demographic, lifestyle, and clinical confounders, most indicators—including RFM, BRI, VAI, LAP, WHtR, WWI, Conicity Index, and BMI—showed significant positive links to GDM. Notably, RFM demonstrated better predictive power for GDM history, slightly outperforming BMI. Nonlinear threshold effects were found for VAI, LAP, and BMI, with a significant interaction between alcohol use and the RFM-GDM link. Overall, these findings highlight the potential of new metrics, especially RFM, to surpass BMI in identifying women at higher GDM risk, thus offering valuable insights for future risk assessment methods.

The main findings of this study align closely with extensive previous research connecting obesity with GDM, while offering a more detailed view. Obesity, especially central obesity, is a well-known risk factor for developing GDM [30, 31]. The main pathophysiological mechanism involves 'lipotoxicity' caused by visceral fat buildup. Excess visceral adipose tissue releases non-esterified fatty acids, pro-inflammatory cytokines such as TNF-α and IL-6, and adipokines, including decreased adiponectin and increased resistin. Collectively, these factors contribute to peripheral insulin resistance and β-cell dysfunction, which disturb normal glucose regulation during pregnancy [32-34]. The current findings significantly broaden the traditional understanding, confirming that, beyond the usual BMI metric, various new composite indicators—such as BRI, WHtR, WWI, and the Conicity Index—that better reflect fat distribution, as well as measures like VAI and LAP that assess metabolic function, are independently and reliably linked to a higher risk of GDM.

The strong correlation between BRI and GDM warrants attention. This aligns with the results from Shi *et al*. in the U.S. population, where BRI was identified as an independent risk factor for GDM [35]. The core reasoning behind this method is that BRI serves as a dependable proxy for visceral obesity, a key factor in metabolic problems. As a result, it effectively detects the “unhealthy obesity” condition, which is strongly linked to insulin resistance [36]. In contrast, RFM showed the highest predictive accuracy in this study, probably due to its distinctive design. RFM calculates total body fat percentage using a solid mathematical model that relies on the ratio of height to waist circumference, and its formula does not directly include body weight. [10, 19]. This method allows RFM to effectively exclude interference from elevated BMI values resulting from high muscle mass (such as “muscle-type obesity”), potentially providing a more accurate measure of fat burden linked to metabolic risk [19, 37]. A study utilizing NHANES data further showed that RFM offers more precise estimates of body fat compared to BMI. [38]. Our findings suggest that RFM is superior to the traditional BMI in assessing GDM risk, a condition closely linked to metabolic disorders. It shows significant potential as a simple, low-cost, and effective marker. This metric is especially useful for large population screenings and initial risk evaluations in resource-constrained environments.

It is noteworthy that the prevalence of GDM and the predictive efficacy of obesity indicators may vary across racial populations. Research conducted on Asian populations has identified a substantial positive correlation between BMI and WHtR with the risk of GDM [39]. Research has demonstrated a close association between RFM and WHtR with type 2 diabetes among European women [40]. Multiple studies have indicated substantial disparities in abdominal fat storage patterns across ethnic groups. These variations in body fat distribution may consequently result in different optimal cutoff values [41-43]. The present study, grounded in a nationally representative multiethnic sample from the United States (comprising non-Hispanic whites, non-Hispanic blacks, Mexicans, *etc*.), has yielded results with robust generalizability. Nevertheless, prospective validation of optimal thresholds and clinical applicability across these indicators in distinct racial cohorts remains imperative.

This study found no significant link between ABSI and GDM, likely due to its original purpose. ABSI was originally created to evaluate the relationship between body shape and mortality risk, accounting for BMI, specifically highlighting trunk fat distribution rather than total fat mass. [15]. As a result, its ability to predict GDM might be restricted, since GDM is strongly associated with overall obesity and metabolic issues. This indicates that, when assessing various health outcomes, choosing the most relevant obesity indicators that accurately represent their underlying pathophysiological processes is crucial.

Subgroup analyses showed an interaction between alcohol consumption and the link between RFM and GDM. For participants who drank alcohol, the positive relationship between RFM and GDM was stronger. This may be because alcohol can affect lipid metabolism and visceral fat buildup, which, together with obesity, may worsen insulin resistance and increase the obesity risk indicated by RFM. [44, 45]. This finding highlights the need to include individual lifestyle factors, like alcohol intake, in future obesity and GDM studies and interventions.

However, the study's design, which excluded some samples during screening due to missing key variables, may have introduced selection bias, potentially compromising the study's external validity. To assess its impact, a thorough analysis of the missing patterns was conducted, along with sensitivity analyses using multiple imputation methods. The findings indicated that the imputed effect estimates showed high concordance with those from the full-case analysis. The direction and significance of the associations between obesity indicators and GDM remained unchanged, suggesting that missing data had a negligible impact on the main conclusions. Furthermore, the weighted variables provided by NHANES partially mitigated bias from non-random missingness. However, the prevailing methodologies are inadequate in addressing bias when missing data is associated with unobserved factors. Furthermore, there remains the potential for residual confounding, as certain critical variables (*e.g.*, pre-pregnancy weight, dietary patterns) were not incorporated into the analysis. Subsequent studies should prioritize optimizing data collection procedures to further substantiate the robustness of these findings.

## 5 STUDY LIMITATIONS

This study has some limitations. First, its cross-sectional design means causality between obesity indicators and GDM cannot be established, underscoring the need for future prospective cohort studies to confirm predictive ability. Second, GDM history was self-reported, which could lead to recall bias. Moreover, the lack of diagnostic details, like gestational age and severity, restricted more detailed subgroup analyses. Future research should combine clinical records with standardized testing. Third, even after adjusting for many covariates, some confounders, such as genetic factors, dietary patterns, and gestational weight gain, were not fully controlled. Gathering more detailed data in future studies is important. Lastly, since the study was conducted only in the United States, the results need validation before applying them to other populations, such as Asian or European groups.

## CONCLUSION

This study, based on NHANES data, systematically compares, for the first time, the associations and predictive efficacy of nine obesity-related indicators (including traditional BMI and novel measures such as RFM, BRI, VAI, LAP, *etc*.) with a history of GDM. The following key findings emerged from the analysis: Except for ABSI, all eight indicators showed a positive, independent correlation with GDM. RFM exhibited the strongest predictive value for GDM, significantly outperforming traditional BMI. Furthermore, nonlinear threshold effects were observed between VAI, LAP, and BMI with GDM, indicating heightened risk increases within specific ranges. The association between RFM and GDM was significantly modified by alcohol consumption status, suggesting that lifestyle factors may play a crucial role in the relationship between obesity and GDM. The primary contribution of this study lies in its comprehensive evaluation of the value of multiple novel obesity indicators for identifying GDM risk using a nationally representative sample. The RFM model offers a novel instrument for clinical screening, characterized by its simplicity and cost-effectiveness, rendering it particularly well-suited for large-scale population screening initiatives. However, given the study's cross-sectional design, causal inferences should be interpreted with caution. The necessity for future prospective cohort studies is twofold: first, to validate the practical efficacy of RFM in early GDM prediction, and second, to explore race-specific cutoff values. These studies will advance the clinical translation of personalized risk assessment.

## Figures and Tables

**Fig. (1):**
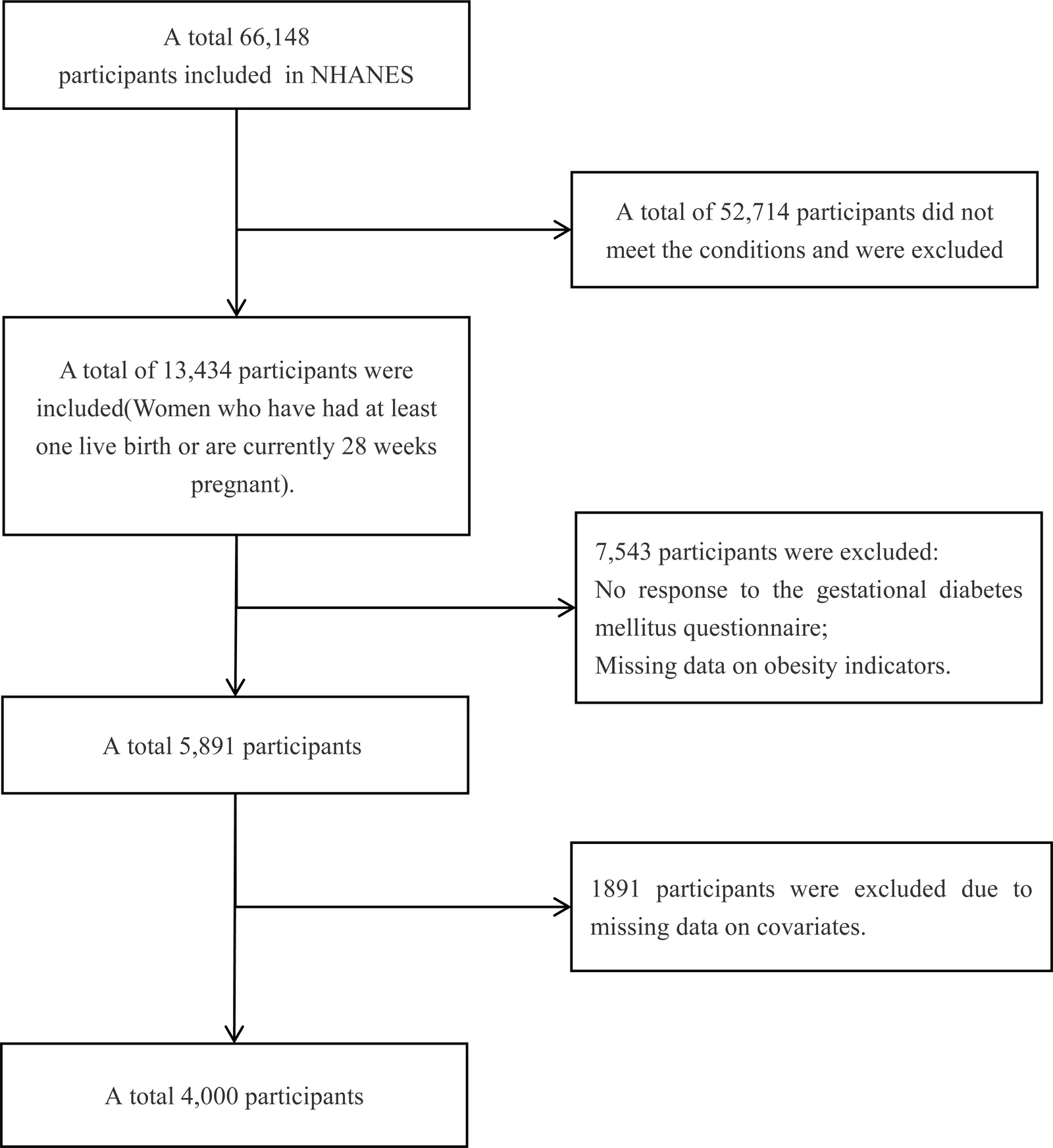
Participant selection flowchart. NHANES, National Health and Nutrition Examination Survey.

**Fig. (2):**
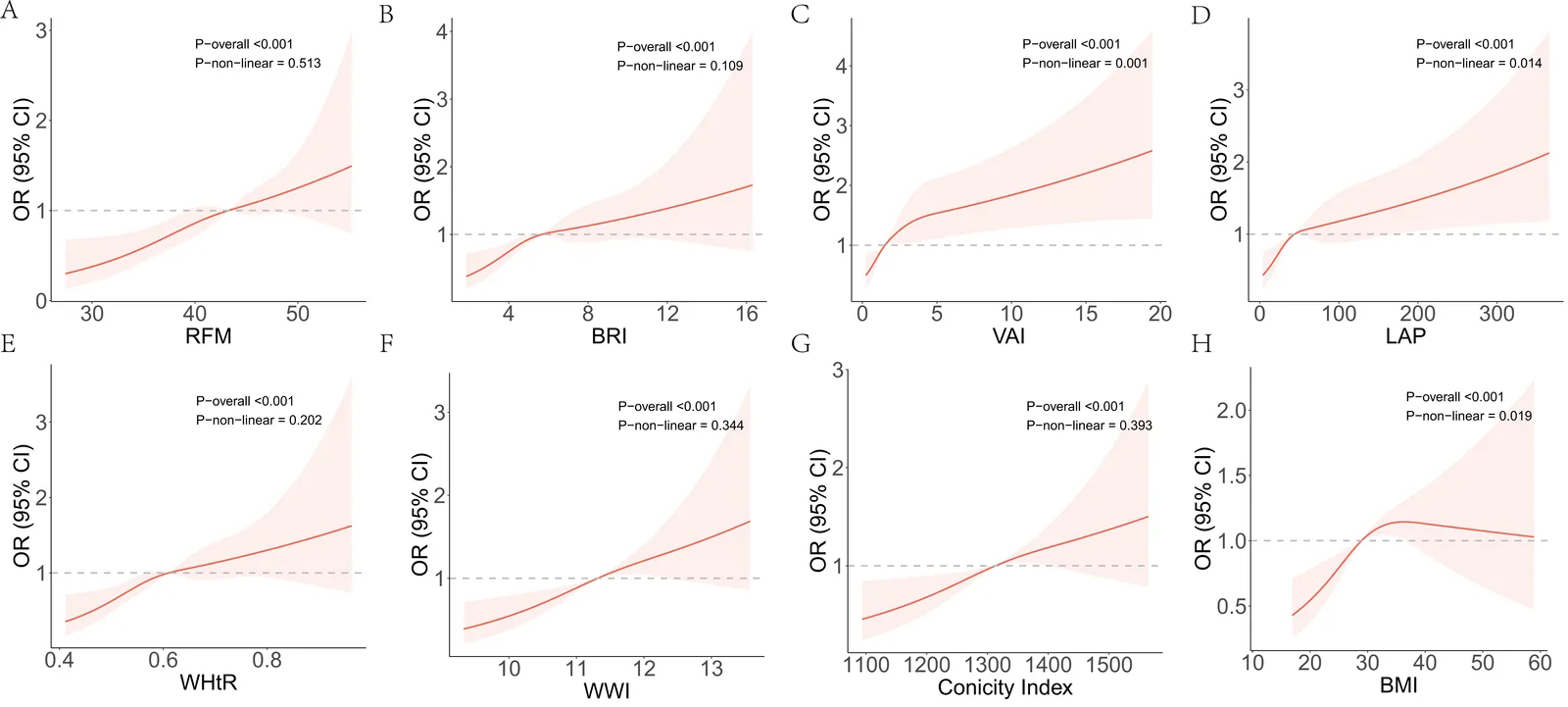
Restricted cubic splines analysis of the relationship between obesity-related indicators and GDM. (**A**) RFM; (**B**) BRI; (**C**) VAI; (**D**) LAP; (**E**) WHtR; (**F**) WWI; (**G**) Conicity Index; (**H**) BMI. Adjusted for age at the time of interview, race, education, PIR, marriage, smoking, alcohol consumption, hypertension, depression, physical activity, infant with birth weight ≥9 lbs or more, and age at first live birth. GDM: gestational diabetes mellitus, RFM: relative fat mass, BRI: body roundness index, VAI: visceral adiposity index, LAP: lipid accumulation product, WHtR: waist to height ratio, WWI: weight-adjusted waist index, BMI: body mass index.

**Fig. (3):**
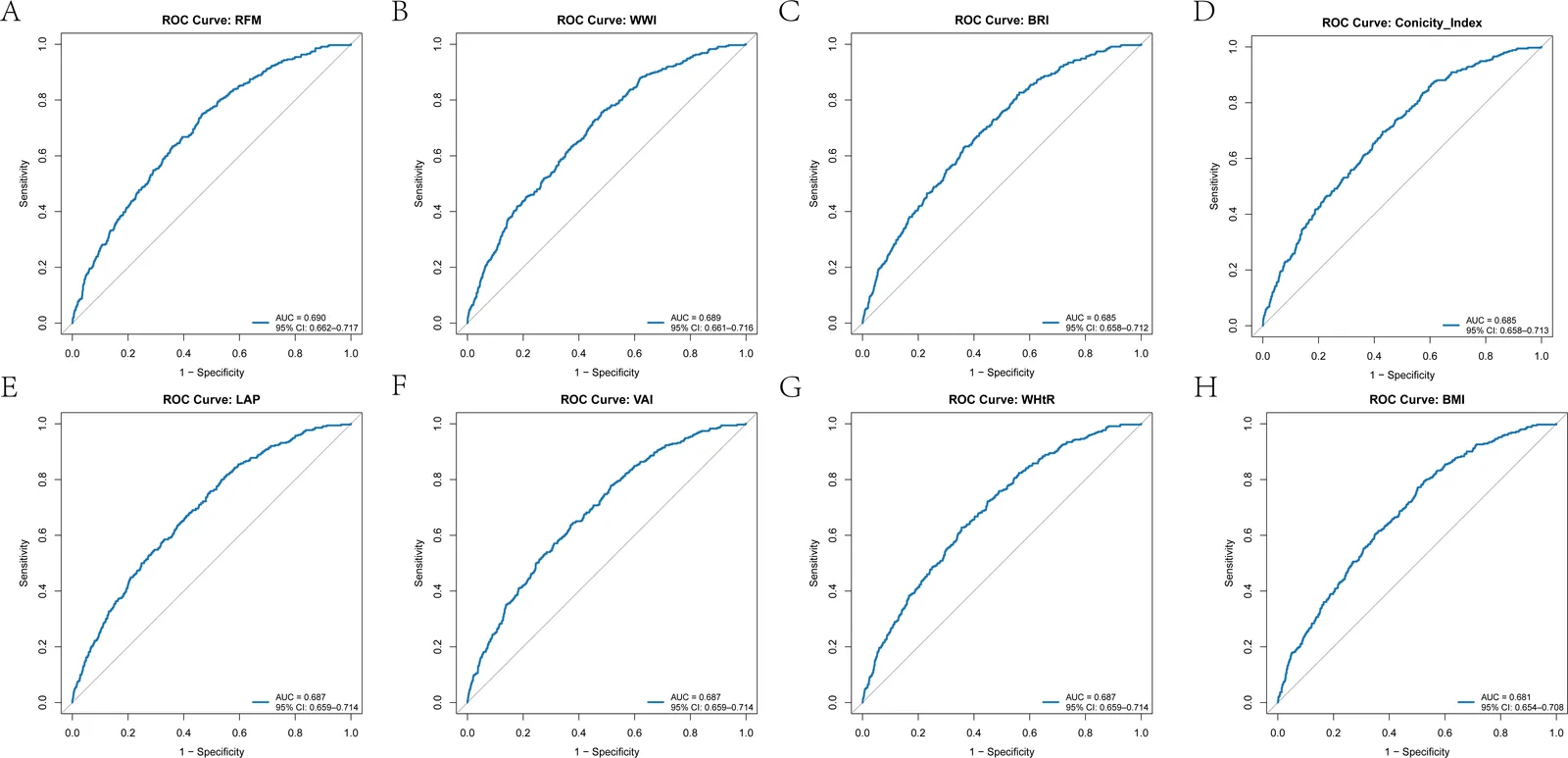
ROC curves for predicting GDM using various obesity-related indicators. (**A**) RFM; (**B**) WWI; (**C**) BRI; (**D**) Conicity Index; (**E**) LAP; (**F**) VAI; (**G**) WHtR; (**H**) BMI. ROC, receiver operating characteristic; GDM: gestational diabetes mellitus, AUC, area under the receiver operating characteristic curve, CI: confidence interval, RFM: relative fat mass, WWI: weight-adjusted waist index, BRI: body roundness index, LAP: lipid accumulation product, VAI: visceral adiposity index, WHtR: waist to height ratio, BMI: body mass index.

**Table 1:** Baseline characteristics of the study population.

**Characteristic**	**Overall**	**Non-GDM**	**GDM**	***p*-value**
Age at the time of interview	50.00 (39.00, 61.00)	51.00 (39.00, 62.00)	44.00 (36.00, 52.00)	<0.001
Age at first live birth	22.00 (19.00, 26.00)	22.00 (19.00, 26.00)	23.00 (20.00, 27.00)	0.067
Race	-	-	-	0.300
Mexican American	650 (8.542%)	581 (8.312%)	69 (11.03%)	-
Non-Hispanic black people	829 (11.77%)	757 (11.86%)	72 (10.79%)	-
Non-Hispanic white people	1,694 (67.24%)	1,564 (67.55%)	130 (63.79%)	-
Other Hispanic	460 (5.793%)	414 (5.682%)	46 (6.993%)	-
Other races	367 (6.658%)	332 (6.590%)	35 (7.393%)	-
Education	-	-	-	0.300
<High school diploma	1,036 (17.02%)	938 (16.66%)	98 (20.83%)	-
>High school diploma	2,050 (58.79%)	1,858 (58.88%)	192 (57.82%)	-
High school diploma/equivalent	914 (24.19%)	852 (24.45%)	62 (21.36%)	-
PIR	2.61 (1.31, 4.67)	2.67 (1.31, 4.74)	2.39 (1.31, 3.97)	0.120
Marriage	-	-	-	0.130
Married/Living with partner	2,389 (67.47%)	2,152 (66.95%)	237 (73.04%)	-
Never married	366 (6.935%)	337 (7.107%)	29 (5.071%)	-
Widowed/Divorced/Separated	1,245 (25.60%)	1,159 (25.94%)	86 (21.89%)	-
Smoking	-	-	-	0.500
Current smoker	737 (18.61%)	661 (18.49%)	76 (19.91%)	-
Former smoker	799 (22.60%)	735 (22.91%)	64 (19.27%)	-
Never smoker	2,464 (58.79%)	2,252 (58.60%)	212 (60.81%)	-
Alcohol consumption	-	-	-	0.110
No	1,723 (36.41%)	1,566 (35.93%)	157 (41.63%)	-
Yes	2,277 (63.59%)	2,082 (64.07%)	195 (58.37%)	-
Physical activity	-	-	-	0.700
Meeting guidelines	2,093 (57.18%)	1,908 (57.07%)	185 (58.36%)	-
Not meeting guidelines	1,907 (42.82%)	1,740 (42.93%)	167 (41.64%)	-
Depression	-	-	-	<0.001
No	3,513 (89.44%)	3,231 (90.15%)	282 (81.81%)	-
Yes	487 (10.56%)	417 (9.85%)	70 (18.19%)	-
Hypertension	-	-	-	0.200
No	2,000 (55.03%)	1,818 (54.66%)	182 (59.02%)	-
Yes	2,000 (44.97%)	1,830 (45.34%)	170 (40.98%)	-
Infant with birth weight ≥9 lbs	-	-	-	0.038
No	3,249 (80.87%)	2,987 (81.45%)	262 (74.53%)	-
Yes	751 (19.13%)	661 (18.55%)	90 (25.47%)	-
RFM	42.63 (38.66, 46.30)	42.52 (38.60, 46.23)	44.10 (40.03, 47.18)	0.007
WWI	11.19 (10.67, 11.78)	11.19 (10.66, 11.77)	11.19 (10.77, 11.85)	0.200
BRI	5.41 (4.05, 7.20)	5.37 (4.03, 7.16)	6.05 (4.47, 7.73)	0.007
ABSI	0.81 (0.05)	0.81 (0.05)	0.81 (0.05)	0.800
ABSI^a^	-0.05 (0.97)	-0.05 (0.97)	-0.07 (0.93)	0.800
Conicity Index	1,306.12 (1,249.41, 1,371.08)	1,305.53 (1,248.34, 1,370.48)	1,311.86 (1,261.37, 1,382.79)	0.200
LAP	42.38 (23.71, 74.20)	41.69 (23.39, 73.36)	49.53 (28.91, 85.65)	0.010
VAI	1.46 (0.89, 2.48)	1.43 (0.88, 2.44)	1.76 (1.04, 2.83)	<0.001
WHtR	0.60 (0.54, 0.67)	0.60 (0.53, 0.67)	0.63 (0.56, 0.69)	0.007
Standing height (cm)	161.89 (6.75)	161.94 (6.78)	161.32 (6.35)	0.300
Waist circumference (cm)	96.60 (86.70, 108.50)	96.50 (86.40, 108.30)	100.90 (89.50, 111.70)	0.011
Weight (kg)	74.20 (63.70, 89.60)	73.80 (63.50, 88.90)	78.30 (67.20, 95.80)	0.014
BMI (kg/m^2^)	28.40 (24.28, 33.80)	28.27 (24.18, 33.60)	30.40 (25.60, 35.60)	0.004
Direct HDL-cholesterol (mmol/L)	1.47 (1.22, 1.76)	1.47 (1.24, 1.78)	1.34 (1.09, 1.55)	<0.001
Triglyceride (mmol/L)	1.08 (0.75, 1.63)	1.07 (0.73, 1.63)	1.16 (0.85, 1.68)	0.061

**Notes:** Categorical variables are expressed as n(%), and continuous variables are expressed as mean (SD) or median [IQR]; n is not weighted; and n(%), mean, SD and median (IQR] are weighted. **Abbreviations:** GDM: gestational diabetes mellitus, BMI: body mass index, PIR: poverty-to-income ratio, RFM: relative fat mass, ABSI: a body shape index, ABSI^a^: the standardized ABSI represented as ABSI^a^, WWI: weight-adjusted waist index, BRI: body roundness index, LAP: lipid accumulation product, VAI: visceral adiposity index, WHtR: waist to height ratio, SD: standard deviation, IQR, interquartile range.

**Table 2:** Relationship between obesity-related indicators and GDM.

**Participants**	**Model 1**	-	**Model 2**	-	**Model 3**	-
	**OR (95%CI)**	***p*-value**	**OR (95%CI)**	***p*-value**	**OR (95%CI)**	***p*-value**
**RFM**	-	-	-	-	-	-
**Continuous**	1.04(1.01, 1.08)	0.004	1.06(1.03, 1.09)	<0.001	1.05(1.02, 1.09)	0.002
**Classification**						
T1	ref.	-	ref.	-	ref.	-
T2	1.35(0.88, 2.07)	0.200	1.55(0.97, 2.48)	0.065	1.52(0.93, 2.47)	0.094
T3	1.64(1.11, 2.41)	0.014	1.92(1.26, 2.93)	0.003	1.71(1.14, 2.58)	0.010
**BRI**	-	-	-	-	-	-
**Continuous**	1.09(1.03, 1.14)	0.001	1.12(1.06, 1.18)	<0.001	1.10(1.04, 1.16)	<0.001
**Classification**						
T1	ref.	-	ref.	-	ref.	-
T2	1.35(0.88, 2.07)	0.200	1.55(0.97, 2.48)	0.065	1.52(0.93, 2.47)	0.094
T3	1.64(1.11, 2.41)	0.014	1.92(1.26, 2.93)	0.003	1.71(1.14, 2.58)	0.010
**VAI**	-	-	-	-	-	-
**Continuous**	1.07(1.03, 1.11)	0.002	1.08(1.03, 1.14)	0.004	1.07(1.02, 1.13)	0.009
**Classification**						
T1	ref.	-	ref.	-	ref.	-
T2	1.32(0.85, 2.06)	0.200	1.43(0.90, 2.29)	0.130	1.45(0.90, 2.33)	0.120
T3	1.74(1.21, 2.50)	0.003	2.09(1.38, 3.15)	<0.001	1.98(1.26, 3.10)	0.003
**LAP**	-	-	-	-	-	-
**Continuous**	1.37(1.15, 1.64)	<0.001	1.53(1.23, 1.90)	<0.001	1.42(1.14, 1.76)	0.002
**Classification**						
T1	ref.	-	ref.	-	ref.	-
T2	1.53(1.04, 2.25)	0.030	1.81(1.20, 2.70)	0.005	1.75(1.15, 2.67)	0.009
T3	1.48(0.99, 2.19)	0.051	1.88(1.22, 2.88)	0.004	1.71(1.09, 2.70)	0.021
**WHtR**	-	-	-	-	-	-
**Continuous**	8.66(2.27, 32.9)	0.002	17.6(4.21, 73.7)	<0.001	11.7(2.76, 49.8)	0.001
**Classification**						
T1	ref.	-	ref.	-	ref.	-
T2	1.35(0.88, 2.07)	0.200	1.55(0.97, 2.48)	0.065	1.52(0.93, 2.47)	0.094
T3	1.64(1.11, 2.41)	0.014	1.92(1.26, 2.93)	0.003	1.71(1.14, 2.58)	0.010
**ABSI^a^**	-	-	-	-	-	-
**Continuous**	0.98(0.85, 1.12)	0.700	1.14(0.98, 1.32)	0.100	1.12(0.96, 1.31)	0.140
**Classification**						
T1	ref.	-	ref.	-	ref.	-
T2	1.14(0.78, 1.68)	0.500	1.33(0.91, 1.94)	0.140	1.29(0.89, 1.88)	0.200
T3	0.97(0.67, 1.40)	0.900	1.33(0.92, 1.92)	0.130	1.29(0.89, 1.88)	0.200
**WWI**	-	-	-	-	-	-
**Continuous**	1.19(1.01, 1.39)	0.034	1.48(1.25, 1.76)	<0.001	1.42(1.20, 1.69)	<0.001
**Classification**						
T1	ref.	-	ref.	-	ref.	-
T2	1.16(0.77, 1.75)	0.500	1.39(0.91, 2.14)	0.130	1.37(0.90, 2.07)	0.140
T3	1.22(0.85, 1.74)	0.300	1.77(1.24, 2.53)	0.002	1.62(1.14, 2.31)	0.007
**Conicity Index**	-	-	-	-	-	-
**Continuous**	1.12(0.99, 1.28)	0.077	1.3(1.14, 1.49)	<0.001	1.25(1.09, 1.44)	0.002
**Classification**						
T1	ref.	-	ref.	-	ref.	-
T2	1.57(1.04, 2.38)	0.032	1.86(1.23, 2.82)	0.004	1.76(1.17, 2.64)	0.007
T3	1.26(0.88, 1.82)	0.200	1.71(1.20, 2.44)	0.004	1.54(1.09, 2.18)	0.014
**BMI**	-	-	-	-	-	-
**Continuous**	1.03(1.01, 1.05)	0.003	1.03(1.01, 1.05)	0.003	1.02(1.00, 1.05)	0.024
**Classification**						
T1	ref.	-	ref.	-	ref.	-
T2	1.15(0.75, 1.78)	0.500	1.25(0.80, 1.96)	0.300	1.23(0.78, 1.93)	0.400
T3	1.75(1.17, 2.61)	0.007	1.84(1.19, 2.83)	0.007	1.65(1.07, 2.55)	0.024

**Note:** Model 1: Non-adjusted; Model 2: Adjusted for age at the time of interview, race, education, PIR and marriage; Model 3: Adjusted for age at the time of interview, race, education, PIR, marriage, smoking, alcohol consumption, hypertension, depression, physical activity, infant with birth weight ≥9 lbs, and age at first live birth. **Abbreviations:** GDM: gestational diabetes mellitus, OR: odds ratio, CI: confidence interval, BMI: body mass index, RFM: relative fat mass, ABSI: a body shape index, ABSI^a^: the standardized ABSI represented as ABSI^a^, WWI: weight-adjusted waist index, BRI: body roundness index, LAP: lipid accumulation product, VAI: visceral adiposity index, WHtR: waist to height ratio.

**Table 3:** AUC values of obesity-related indicators.

**Variables**	**AUC**	**95%CI**	***P***
BMI	0.681	0.654–0.708	-
RFM	0.690	0.662–0.717	<0.001
WWI	0.689	0.661–0.716	0.082
BRI	0.685	0.658–0.712	0.036
ABSI^a^	0.680	0.652–0.707	0.798
Conicity Index	0.685	0.658–0.713	0.289
LAP	0.687	0.659–0.714	0.114
VAI	0.687	0.659–0.714	0.202
WHtR	0.687	0.659–0.714	0.006

**Abbreviations:** AUC: area under the curve, CI: confidence interval, BMI: body mass index, RFM: relative fat mass, ABSI: a body shape index, ABSI^a^: the standardized ABSI represented as ABSI^a^, WWI: weight-adjusted waist index, BRI: body roundness index, LAP: lipid accumulation product, VAI: visceral adiposity index, WHtR: waist to height ratio.

**Table 4:** Subgroup analyses of the associations between RFM and GDM.

**Participants**	**OR (95%CI)**	***P*-value**	***P* for Interaction**
Race	-	-	0.649
Mexican American	1.03(0.96, 1.11)	0.400	-
Non-Hispanic black people	1.06(1.00, 1.13)	0.034	-
Non-Hispanic white people	1.06(1.01, 1.11)	0.021	-
Other Hispanic	1.08(0.97, 1.19)	0.140	-
Other races	1.09(0.98, 1.22)	0.120	-
Education	-	-	0.301
<High school diploma	1.04(0.98, 1.10)	0.200	-
>High school diploma	1.07(1.03, 1.11)	<0.001	-
High school diploma/equivalent	1.02(0.95, 1.10)	0.500	-
Marriage	-	-	0.255
Married/Living with partner	1.07(1.02, 1.11)	0.002	-
Never married	1.03(0.95, 1.11)	0.500	-
Widowed/Divorced/Separated	1.02(0.96, 1.09)	0.400	-
Smoking	-	-	0.098
Current smoker	0.99(0.93, 1.06)	0.800	-
Former smoker	1.09(1.01, 1.17)	0.027	-
Never smoker	1.07(1.04, 1.11)	<0.001	-
Alcohol consumption	-	-	0.039
No	1(0.97, 1.04)	0.800	-
Yes	1.09(1.04, 1.14)	<0.001	-
Physical activity	-	-	0.495
Meeting guidelines	1.06(1.01, 1.10)	0.010	-
Not meeting guidelines	1.04(0.99, 1.08)	0.089	-
Depression	-	-	0.918
No	1.05(1.01, 1.09)	0.006	-
Yes	1.06(1.00, 1.12)	0.074	-
Infant with birth weight ≥9 lbs	-	-	0.557
No	1.05(1.01, 1.09)	0.014	-
Yes	1.07(1.01, 1.14)	0.032	-
Hypertension	-	-	0.079
No	1.04(1.00, 1.08)	0.071	-
Yes	1.09(1.04, 1.14)	<0.001	-

**Note:** Adjusted for age at the time of interview, race, education, PIR, marriage, smoking, alcohol consumption, hypertension, depression, physical activity, infant with birth weight ≥9 lbs, and age at first live birth. **Abbreviations:** RFM: relative fat mass, GDM: gestational diabetes mellitus, OR: odds ratio, CI: confidence interval.

## Data Availability

The National Health and Nutrition Examination Survey dataset is publicly available at the National Center for Health Statistics of the Centers for Disease Control and Prevention (https://www.cdc.gov/nchs/nhanes/).

## LIST OF ABBREVIATIONS

ABSI: A Body Shape Index
ABSIa: The Standardized ABSI
AUC: Area Under the Curve
BMI: Body Mass Index
BRI: Body Roundness Index
CI: Confidence Interval
GDM: Gestational Diabetes Mellitus
HDL-C: High-Density Lipoprotein Cholesterol
IL-6: Interleukin-6
IQR: Interquartile Range
LAP: Lipid Accumulation Product
MET: Metabolic Equivalent of Task
NHANES: National Health and Nutrition Examination Survey
OR: Odds Ratio
PA: Physical Activity
PHQ-9: Patient Health Questionnaire-9
PIR: Poverty-to-Income Ratio
RCS: Restricted Cubic Splines
RFM: Relative Fat Mass
ROC: Receiver Operating Characteristic
TG: Triglyceride
TNF-α: Tumor Necrosis Factor-alpha
VAI: Visceral Adiposity Index
WC: Waist Circumference
WHtR: Waist-to-Height Ratio
WWI: Weight-Adjusted Waist Index
