# Supplementary material for: Association of Obesity Indicators with Gestational Diabetes: A Comparative Study from NHANES
Source: Endocr Metab Immune Disord Drug Targets. 2026 Jun 10;26:E18715303466539. doi: 10.2174/0118715303466539260525105816 (PMC13659190; doi:10.2174/0118715303466539260525105816)
Supplement: Supplementary file 1 [file EMIDDT-26-E18715303466539_SD1.pdf]

Supplementary Material

Association of Obesity Indicators with Gestational Diabetes: A Comparative Study from NHANES

Jing Tang[1,*]

[1]*Shanghai Key Lab of Female Reproductive Endocrine Related Diseases, Obstetrics & Gynecology Hospital of Fudan University, Shanghai Key Lab of Reproduction and Development, Shanghai, China*

Table S1. Relationship between obesity-related indicators and GDM (unweighted).

Participants	Model 1		Model 2		Model 3	
	OR (95%CI)	*p*-value	OR (95%CI)	*p*-value	OR (95%CI)	*p*-value
**LAP**	1.38(1.18, 1.62)	<0.001	1.53(1.28, 1.83)	<0.001	1.39(1.18, 1.66)	<0.001
**RFM**	1.05(1.02, 1.07)	<0.001	1.06(1.04, 1.09)	<0.001	1.05(1.03, 1.08)	<0.001
**BRI**	1.08(1.04, 1.13)	<0.001	1.11(1.06, 1.16)	<0.001	1.09(1.04, 1.14)	<0.001
**VAI**	1.06(1.03, 1.10)	<0.001	1.07(1.04, 1.11)	<0.001	1.06(1.03, 1.10)	<0.001
**WHtR**	8.54(2.98, 24.2)	<0.001	16.9(5.71, 49.5)	<0.001	10.1(3.22, 31.6)	<0.001
**ABSI[a]**	1.01(0.90, 1.12)	0.903	1.21(1.07, 1.37)	0.003	1.18(1.05, 1.34)	0.008
**WWI**	1.2(1.04, 1.37)	0.010	1.53(1.32, 1.78)	<0.001	1.47(1.25, 1.71)	<0.001
**Conicity Index**	1.15(1.03, 1.28)	0.013	1.35(1.20, 1.52)	<0.001	1.28(1.14, 1.45)	<0.001
**BMI**	1.03(1.01, 1.04)	<0.001	1.03(1.01, 1.04)	<0.001	1.02(1.00, 1.04)	0.011

Note: *Model 1:* Unadjusted; *Model 2:* Adjusted for age at the time of interview, race, education, PIR and marriage; *Model 3:* Adjusted for age at the time of interview, race, education, PIR, marriage, smoke, drink, hypertension, depression, PA, infant with birth weight ≥9 lbs, and age at first live birth.

Abbreviations: ABSI[a]: Standardized A Body Shape Index; BMI: Body Mass Index; BRI: Body Roundness Index; CI: Confidence Interval; GDM: Gestational Diabetes Mellitus; LAP: Lipid Accumulation Product; OR: Odds Ratio; PA: Physical Activity; PIR: Poverty-to-Income Ratio; RFM: Relative Fat Mass; VAI: Visceral Adiposity Index; WHtR: Waist-to-Height Ratio; WWI: Weight-Adjusted Waist Index

Table S2. Relationship between obesity-related indicators and GDM.

Participants	Model 3		Model 4	
	OR (95%CI)	*p*-value	OR (95%CI)	*p*-value
**LAP**	1.39(1.18, 1.66)	<0.001	1.60(1.21, 2.12)	0.002
**RFM**	1.05(1.03, 1.08)	<0.001	1.06(1.01, 1.10)	0.008
**BRI**	1.09(1.04, 1.14)	<0.001	1.11(1.04, 1.19)	0.003
**VAI**	1.06(1.03, 1.10)	<0.001	1.09(1.02, 1.17)	0.018
**WHtR**	10.1(3.22, 31.6)	<0.001	15(2.44, 92.0)	0.004
**ABSI**[a]	1.18(1.05, 1.34)	0.008	1.1(0.91, 1.32)	0.300
**WWI**	1.47(1.25, 1.71)	<0.001	1.43(1.16, 1.76)	0.001
**Conicity Index**	1.28(1.14, 1.45)	<0.001	1.23(1.04, 1.46)	0.016
**BMI**	1.02(1.00, 1.04)	0.011	1.03(1.00, 1.06)	0.033

Note: *Model 3:* Adjusted for age at the time of interview, race, education, PIR, marriage, smoke, drink, hypertension, depression, PA, infant with birth weight ≥9 lbs, and age at first live birth; *Model 4:* Model 1 + number of vaginal deliveries + contraceptive use + female hormone use + number of deliveries live birth result.

Abbreviations: ABSI[a]: Standardized A Body Shape Index; BMI: Body Mass Index; BRI: Body Roundness Index; CI: Confidence Interval; GDM: Gestational Diabetes Mellitus; LAP: Lipid Accumulation Product; OR: Odds Ratio; PA: Physical Activity; PIR: Poverty-to-Income Ratio; RFM: Relative Fat Mass; VAI: Visceral Adiposity Index; WHtR: Waist-to-Height Ratio; WWI: Weight-Adjusted Waist Index

Table S3. Relationship between obesity-related indicators and GDM.

Participants	Model 1		Model 2		Model 3	
	OR (95%CI)	*p*-value	OR (95%CI)	*p*-value	OR (95%CI)	*p*-value
**RFM**						
**Continuous**	1.04(1.02, 1.07)	<0.001	1.06(1.03, 1.09)	<0.001	1.04(1.02, 1.07)	0.002
**Classification**						
T1	ref.		ref.		ref.	
T2	1.42(0.98, 2.05)	0.062	1.59(1.07, 2.37)	0.022	1.47(0.97, 2.20)	0.066
T3	1.59(1.17, 2.17)	0.004	1.97(1.40, 2.78)	<0.001	1.62(1.15, 2.30)	0.007
**BRI**						
**Continuous**	1.08(1.03, 1.12)	<0.001	1.11(1.06, 1.16)	<0.001	1.08(1.03, 1.13)	0.002
**Classification**						

T1	ref.		ref.		ref.	
T2	1.42(0.98, 2.05)	0.062	1.59(1.07, 2.37)	0.022	1.47(0.97, 2.20)	0.066
T3	1.59(1.17, 2.17)	0.004	1.97(1.40, 2.78)	<0.001	1.62(1.15, 2.30)	0.007
**VAI**						
**Continuous**	1.09(1.04, 1.13)	<0.001	1.1(1.05, 1.15)	<0.001	1.08(1.03, 1.14)	0.002
**Classification**						
T1	ref.		ref.		ref.	
T2	1.35(0.90, 2.03)	0.150	1.44(0.95, 2.18)	0.089	1.35(0.88, 2.07)	0.200
T3	1.9(1.39, 2.59)	<0.001	2.18(1.53, 3.11)	<0.001	1.95(1.33, 2.85)	<0.001
**LAP**						
**Continuous**	1.46(1.25, 1.70)	<0.001	1.61(1.34, 1.94)	<0.001	1.46(1.20, 1.78)	<0.001
**Classification**						
T1	ref.		ref.		ref.	
T2	1.51(1.08, 2.11)	0.017	1.77(1.24, 2.53)	0.002	1.66(1.14, 2.41)	0.008
T3	1.68(1.24, 2.29)	0.001	2.10(1.49, 2.97)	<0.001	1.79(1.23, 2.60)	0.003
**WHtR**						
**Continuous**	7.09(2.41, 20.8)	<0.001	15.9(4.84, 52.1)	<0.001	7.42(2.16, 25.5)	0.002
**Classification**						
T1	ref.		ref.		ref.	
T2	1.42(0.98, 2.05)	0.062	1.59(1.07, 2.37)	0.022	1.47(0.97, 2.20)	0.066
T3	1.59(1.17, 2.17)	0.004	1.97(1.40, 2.78)	<0.001	1.62(1.15, 2.30)	0.007
**ABSI**[a]						
**Continuous**	0.94(0.84, 1.06)	0.300	1.1(0.96, 1.26)	0.200	1.09(0.95, 1.25)	0.200
**Classification**						
T1	ref.		ref.		ref.	
T2	1.08(0.79, 1.46)	0.600	1.21(0.90, 1.64)	0.200	1.17(0.86, 1.58)	0.300
T3	0.87(0.64, 1.19)	0.400	1.21(0.87, 1.67)	0.200	1.16(0.83, 1.62)	0.400
**WWI**						
**Continuous**	1.12(0.99, 1.27)	0.074	1.41(1.21, 1.65)	<0.001	1.32(1.13, 1.55)	<0.001
**Classification**						

T1	ref.		ref.		ref.	
T2	1.09(0.77, 1.54)	0.600	1.25(0.87, 1.79)	0.200	1.16(0.81, 1.66)	0.400
T3	1.08(0.80, 1.46)	0.600	1.6(1.17, 2.20)	0.004	1.39(1.02, 1.91)	0.040
**Conicity Index**						
**Continuous**	1.09(0.98, 1.21)	0.110	1.28(1.14, 1.44)	<0.001	1.2(1.06, 1.36)	0.005
**Classification**						
T1	ref.		ref.		ref.	
T2	1.32(0.94, 1.83)	0.100	1.53(1.09, 2.14)	0.014	1.4(1.01, 1.96)	0.047
T3	1.06(0.78, 1.44)	0.700	1.5(1.11, 2.03)	0.010	1.27(0.94, 1.72)	0.120
**BMI**						
**Continuous**	1.03(1.01, 1.04)	<0.001	1.03(1.01, 1.05)	<0.001	1.02(1.00, 1.04)	0.027
**Classification**						
T1	ref.		ref.		ref.	
T2	1.15(0.78, 1.70)	0.500	1.21(0.80, 1.81)	0.400	1.11(0.74, 1.66)	0.600
T3	1.79(1.28, 2.52)	<0.001	1.9(1.32, 2.74)	<0.001	1.57(1.08, 2.27)	0.018

NOTE: *model 1*: Non-adjusted; *model 2:* Adjusted for age at the time of interview, race, education, PIR, and marriage; *model 3:* Adjusted for age at the time of interview, race, education, PIR, marriage, smoking, alcohol consumption, hypertension, depression, PA, any babies weighing 9 lbs or more.

Abbreviations: ABSI[a]: Standardized A Body Shape Index; BMI: Body Mass Index; BRI: Body Roundness Index; CI: Confidence Interval; GDM: Gestational Diabetes Mellitus; LAP: Lipid Accumulation Product; OR: Odds Ratio; PA: Physical Activity; PIR: Poverty-to-Income Ratio; RFM: Relative Fat Mass; VAI: Visceral Adiposity Index; WHtR: Waist-to-Height Ratio; WWI: Weight-Adjusted Waist Index
